# Epidemiology, Modern Diagnostics, and the Management of Mucorales Infections

**DOI:** 10.3390/jof9060659

**Published:** 2023-06-12

**Authors:** David Pham, Annaleise R. Howard-Jones, Rebecca Sparks, Maurizio Stefani, Varsha Sivalingam, Catriona L. Halliday, Justin Beardsley, Sharon C.-A. Chen

**Affiliations:** 1 Centre for Infectious Diseases & Microbiology, Westmead Hospital, Westmead, NSW 2170, Australia; david.pham@health.nsw.gov.au (D.P.);; 2 Centre for Infectious Diseases & Microbiology Laboratory Services, NSW Health Pathology—Institute of Clinical Pathology & Medical Research, Westmead Hospital, Westmead, NSW 2170, Australia; 3 Faculty of Medicine & Health, University of Sydney, Camperdown, NSW 2006, Australia; 4 Sydney Institute for Infectious Diseases, University of Sydney, Sydney, NSW 2006, Australia; 5 Douglass Hanly Moir Pathology, Sydney, NSW 2113, Australia; 6 Westmead Institute for Medical Research, Sydney, NSW 2145, Australia

**Keywords:** mucormycosis, Mucorales, epidemiology, DNA sequencing, diagnostics, antifungal agents

## Abstract

Mucormycosis is an uncommon, yet deadly invasive fungal infection caused by the Mucorales moulds. These pathogens are a WHO-assigned high-priority pathogen group, as mucormycosis incidence is increasing, and there is unacceptably high mortality with current antifungal therapies. Current diagnostic methods have inadequate sensitivity and specificity and may have issues with accessibility or turnaround time. Patients with diabetes mellitus and immune compromise are predisposed to infection with these environmental fungi, but COVID-19 has established itself as a new risk factor. Mucorales also cause healthcare-associated outbreaks, and clusters associated with natural disasters have also been identified. Robust epidemiological surveillance into burden of disease, at-risk populations, and emerging pathogens is required. Emerging serological and molecular techniques may offer a faster route to diagnosis, while newly developed antifungal agents show promise in preliminary studies. Equitable access to these emerging diagnostic techniques and antifungal therapies will be key in identifying and treating mucormycosis, as delayed initiation of therapy is associated with higher mortality.

## 1. Introduction

Mucormycosis is an uncommon but aggressive invasive disease caused by fungi belonging to the order Mucorales, with the most common causative genera being *Rhizopus*, *Mucor*, and *Lichtheimia* [1,2,3]. Mucorales are ubiquitous environmental moulds and rarely cause invasive disease in immunocompetent hosts; however, patients with mucormycosis typically have predisposing conditions, such as diabetes mellitus, malignancy, haematopoietic or solid-organ transplantation, and, most recently, concurrent or recent COVID-19 infection [4,5,6]. The patient population at risk for mucormycosis continues to expand due to increasing metabolic disease and the use of immunosuppressive therapies, leading to an increasing incidence of mucormycosis worldwide. Mucorales has also been well-recognised to cause outbreaks within healthcare settings or following natural disasters [7].

Infection is typically acquired through the inhalation of spores into the sinuses or lung of the susceptible host, with subsequent spread to contiguous structures or dissemination via angioinvasion, depending on the host immune response. The most common presentations are rhino-orbital-cerebral mucormycosis (ROCM), typically associated with diabetes mellitus, and pulmonary mucormycosis, which is the most common manifestation in haematology patients; gastrointestinal (GI), cutaneous and soft tissue, and disseminated mucormycosis also occur [1,2]. The diagnosis of mucormycosis is reliant on the identification of fungal angioinvasion on histopathology, as well as culture and culture-independent microbiological methods, with imaging used to support the diagnosis [3,8]. Timely diagnosis is essential for maximising survival through early medical and surgical interventions [3,9]. Even with early recognition and management, the prognosis remains poor, with mortality estimated at 60% for ROCM and 57% for pulmonary mucormycosis in cases published worldwide [10,11].

Mucorales were recently designated as high-priority pathogens by the WHO, receiving this ranking on account of challenges with drug susceptibility and diagnosis [12]. Within this narrative review, we outline the emerging literature in the epidemiology of Mucorales infections, with a focus on newer risks and outbreaks. We also provide an update on the taxonomy of these fungi to provide clarity on causative pathogens; discuss diagnostic methods, including whole-genome sequencing (WGS) approaches; and review key management strategies, including new antifungal agents.

## 2. Taxonomy of Mucorales

One of the most ancient groups of fungi, the order Mucorales, is characterised by abundant thin-walled aseptate mycelium. Taxonomy was traditionally based upon microscopic morphology and mating experiments, with these organisms classified in the phylum Zygomycota. However, molecular phylogenetic studies of multiple gene regions, including the nuclear ribosomal RNA subunits (18*S*, 5.8*S* and 28*S*), RNA polymerase subunits (*RPB1* and *RPB2*), elongation factor 1-alpha, and α- and β-tubulins, indicated that Zygomycota was polyphyletic [13]. The phylum Zygomycota was abandoned following further phylogenetic analysis in favour of two phyla: Mucoromycota and Zoopagomycota. The Mucoromycota consist of three subphyla: Glomeromycotina, Mortierellomycotina, and Mucoromycotina, of which the latter include the orders Mucorales, Umbelopsidales, and Endogenales [14,15].

Within Mucorales, the structure may be classified at the family level, using four molecular markers: (i) large and (ii) small subunit of the ribosomal DNA, (iii) the partial actin gene, and (iv) the partial gene for elongation factor 1-alpha [16]. Mucorales fungi reproduce asexually by means of single-celled sporangiospores produced endogenously in either sporangia (multi-spored), sporangiola (with 1 or few spores), or merosporangia (spores in rows). Sexual reproduction occurs via the fusion of two opposed suspensors originating from different hyphae to form a zygospore [17]. Additional morphological characteristics are now not considered to be taxonomically relevant [15].

The family structure including the 11 genera (and at least 39 species) of Mucorales described as human pathogens is summarised in Table 1 [15,16]. *Rhizopus* and *Mucor*, followed by *Lichtheimia* and *Apophysomyces*, are the main pathogenic genera; and *Rhizopus arrhizus*, *Rhizopus microsporus*, and *Mucor circinelloides* the main species [15,18]. Historically, *M. circinelloides* was divided into four formae, namely f. *circinelloides*, f. *griseocyanus*, f. *janssenii*, and f. *lusitanicus*, but these now represent the species *M. circinelloides* sensu stricto, *M. griseocyanus*, *M. janssenii*, and *M. lusitanicus*, respectively; and the additional species *M. ramosissimus*, *M. variicolumellatus*, and *M. velutinosus* make up the *M. circinelloides* complex known to cause human infections [18]. There is ongoing debate regarding the species concept of *R. arrhizus* (syn *R. oryzae*); in this review, two varieties are referred to—*R. arrhizus* var. *arrhizus* and *R. arrhizus* var. *delemar* [15,19].

## 3. Epidemiology of Mucormycosis

### 3.1. Update on Epidemiology: Burden and Causative Pathogens

The WHO reports that the burden of disease caused by Mucorales is currently unknown due to variable reporting strategies and incomplete surveillance data, as mucormycosis is not a reportable disease in most jurisdictions [12]. Other than two systematic reviews of mucormycosis by Roden et al. (929 cases from 1940 to 2003 [1] and by Jeong et al. (851 cases from 2000 to 2017) [2], most data are presented as case series or reports, which typically focus on national or regional epidemiology or are confined to in specific populations [5,21,22,23,24,25,26,27,28,29,30,31,32]. Systematic reviews have also been undertaken for specific clinical manifestations, such as cutaneous, GI, and pulmonary mucormycosis [11,33,34,35].

Despite imperfect epidemiological data, there appeared to be an increasing incidence of mucormycosis worldwide, even prior to the COVID-19 pandemic [1,23,36]. In part, this is attributed to improved recognition and diagnosis, as well as increasing prevalence of risk factors such as diabetes mellitus and immunosuppression [37]. A French nationwide population study revealed an increase from 0.7 to 1.2 cases per million population per year between 1997 and 2006 [36], while the incidence increased 2.5-fold between 2008 and 2018 in an Iranian case series [23]. 

Globally, the highest reported incidence of mucormycosis comes from India, around 70× higher than elsewhere [38,39]. This has been attributed, in part, to high rates of poorly controlled diabetes mellitus. The overall incidence increased from an estimated 25 cases per year (from 1990–2007) to 89 cases per year (2013–2015) in one tertiary hospital [24], while a series of cross-sectional studies from Chakrabarti et al. showed an increase from 12.9 cases per year (1990–1999) to 50 cases per year at another tertiary hospital (2006–2007) [40,41,42]. A computational model estimated a mean of 171,504 cases (95% CI 147,688–195,777) and 65,500 deaths (38.2% mortality) per year in India prior to COVID-19. Finally, the estimated incidence was 14 cases per 100,000 population per year in India and Pakistan, compared to 0.05–9.5 cases per 100,000 population per year globally [39,43]. 

*Rhizopus* (48%), *Mucor* (14%), and *Lichtheimia* (13%) spp. are responsible for the majority of mucormycosis cases worldwide, but there is significant regional variation in causative pathogens [2]. These variations are likely attributable to endemicity in the environment, as well as pathophysiological factors such as temperature-dependent growth parameters. For instance, *Apophysomyces* cases comprise the second most reported Mucorales infections in India, possibly due to the thermotolerant nature of the fungus and its ubiquitous presence the local environment [44,45]. Conversely, *Apophysomyces* cases have been less commonly described in Africa and Europe, while *Lichtheimia* is responsible for a greater proportion of European cases [2].

Certain Mucorales have associations with specific clinical manifestations. For instance, *Cunninghamella* spp. are more common in patients with pulmonary disease (57% versus 20%, *p* < 0.001) or disseminated disease (33% versus 13%, *p* = 0.002), and mortality associated with *Cunninghamella* infections is significantly higher than that caused by other Mucorales (71% versus 44%, *p* < 0.001) [2]. *Apophysomyces* spp. (65%), *Lichtheimia* spp. (45%), and *Saksenaea* complex (75%) were more commonly isolated in patients with cutaneous disease [2]. 

Several emerging species are notable. *Rhizopus homothallicus* was first noted as the cause of two cases of cavitary pulmonary mucormycosis in 2010 [46]. Subsequent case series from India have also reported *R. homothallicus* causing cutaneous mucormycosis, ROCM [24,47,48,49,50], and CAM [51,52]. *Mucor irregularis* (previously *Rhizomucor variabilis*), first described as causing cutaneous mucormycosis in China [53], has since been reported in China and India to be associated with chronic cutaneous mucormycosis in immunocompetent patients [50,54,55]. *Mucor velutinosus*, first described in 2011, has been implicated in cutaneous and bloodstream disease [56,57,58,59]. *Saksenaea* erythrospora, first described as causing orbital mucormycosis in 2011 [60], is known to cause cutaneous or orbital disease following trauma or surgery, with descriptions from Asia and the Americas [61,62]. Other emerging Mucorales include *Thamnostylum lucknowense* [63], *Cunninghamella arunalokei* [20] and *Lichtheimia ornata* [64] (all ROCM), and *Apophysomyces mexicanus* (cutaneous mucormycosis) [65].

### 3.2. Host Risk Factors

The traditional host risk factors for mucormycosis are well-established and have been detailed in systematic reviews by Roden et al. and Jeong et al. [1,2], as well as other publications within Table 2. These factors both predispose to Mucorales infection and influence the clinical presentation and prognosis of disease [66]. 

#### COVID-19-Associated Mucormycosis

Fungal superinfections are well-recognised in patients with severe COVID-19 or those recovering from the disease. In early 2021, increasing cases of CAM were reported from India, with an estimated 2.1-fold rise in mucormycosis during September–December 2020, compared to September–December 2019 [76]. Factors contributing to this include high background prevalence of mucormycosis in India, high incidence of undiagnosed or poorly controlled diabetes mellitus, COVID-19 immune dysregulation, and the immunosuppressive effect of COVID-19 therapies such as steroids [77,78]. Although at comparatively lower rates of incidence, CAM has been reported globally [79,80].

Several systematic reviews have found that CAM occurs predominantly in males (70–79%), patients with underlying diabetes (78–83%), and/or those receiving corticosteroids (76–79%) [6,77,79,81]. *Rhizopus* spp. were the most implicated pathogens [6]. Most presentations were with ROCM (73–89%), and mortality varied between 30 and 49% [82]. The pathophysiology is thought to involve the interplay of hyperglycaemia, diabetic ketoacidosis, immunosuppression, free iron availability, immune dysregulation, and pulmonary damage from COVID-19 predisposing to tissue invasion [83]. The detailed pathogenesis of CAM is beyond the scope of this review, but the reader is referred to several comprehensive reviews on this topic [84,85,86].

**Figure 1:**
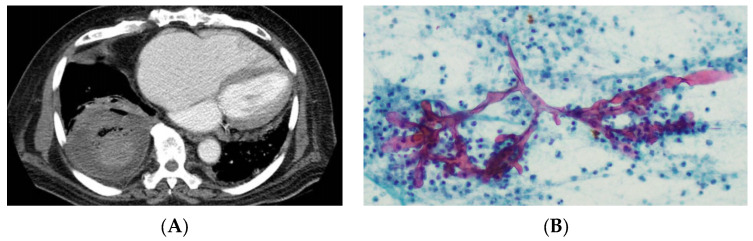
(**A**) Chest computed tomography in a patient with COVID-19 demonstrating a large mass in the right lower lobe of the lung with the beginnings of cavitation subsequently diagnosed as mucormycosis. (**B**) Histopathology demonstrating irregular broad aseptate hyphae (*Rhizopus* spp.) visualized by Periodic Acid Schiff staining on lung biopsy of the mass in panel A.

Major challenges with CAM have included difficulties with diagnosis and with access to effective therapies. The imaging changes of mucormycosis may overlap with pneumonitis due to SARS-CoV-2 or be reminiscent of other invasive fungal superinfection with mass lesions, cavitations, and nodules, thus preventing early recognition (Figure 1A) [87]. Access to invasive sampling of respiratory specimens was also reduced due to COVID-19 infection prevention precautions. Additionally, the increased burden of COVID-19 also reduced healthcare resources that may have otherwise been applied to recognise, diagnosis, and treat CAM more effectively [88]. Notably, in India, supplies of effective antifungal agents were rapidly exhausted during their outbreak, exposing weaknesses in supply chains [89]. Moving forward, a high degree of awareness is required to swiftly diagnose and manage patients with CAM.

### 3.3. Healthcare-Associated Mucormycosis

Although Mucorales cause mostly sporadic infections in the community setting, nosocomial outbreaks are increasingly recognised [7,90,91]. Outbreaks can present with the full range of clinical presentations, including ROCM and disseminated disease. Predisposing factors for disease are similar to sporadic infections (Table 2) [90]. Exposure to mucormycetes can occur throughout the entire healthcare setting, with infections linked to construction [92,93,94]; insufficient ventilation [95,96,97,98]; and contaminated medical devices [99,100,101,102,103,104,105,106], linen [107,108,109,110,111,112], and food [113,114,115,116]. 

Clusters have been described in several hospital settings, including in intensive care units (ICUs) [117], transplantation units as graft-transmitted mucormycosis in liver- and kidney-transplant recipients [118,119,120], and notably in burn units [34,121,122]. Burn patients have extensive disruptions of the physical skin barrier, along with immune dysfunction. A systematic review of 114 patients across 46 studies showed that the most common pathogen was *Mucor* spp. (44%) [34]. Cutaneous infection was the most common clinical syndrome (84%), while only 6% of cases were ROCM. Both an increasing percentage of total body surface area burned and infection in two or more locations were associated with higher mortality [34]. Two outbreaks within burn units were due to the contamination of non-sterile bandages [123,124]. 

Recognition and investigation of healthcare-associated outbreaks can be difficult [125,126], and despite best efforts, epidemiological links can be difficult to establish [127]. Investigations should involve robust hypothesis-testing, the detection of potential healthcare sources, and consideration of environmental testing [125,126]. Molecular methods, including WGS, have emerged to characterise the genomic epidemiology of these outbreaks (see Section 4.8) [128,129,130,131]. “Pseudo-outbreaks” of Mucorales have also been described, and it is important to differentiate true noscomial clusters from environmental contamination or issues resulting from changes in surveillance or testing. For instance, three pseudo-outbreaks of *Rhizopus* spp. infection originated from contaminated non-sterile wooden sticks or tongue depressors [132,133,134]. In each outbreak, at least one patient was treated with amphotericin for presumptive mucormycosis prior to recognition of the contamination. A third outbreak of *M. circinelloides* sensu stricto was initially implicated over two Parisian hospitals, with WGS eventually identifying a high degree of genetic diversity amongst the isolates without a point source [124,129].

### 3.4. Natural Disaster-Associated Mucormycosis

Natural disasters predispose environments to outbreaks of mucormycosis by providing a traumatic portal of entry, whilst disturbed earth or flood waters may carry pathogens, and certain weather patterns have even been hypothesised to induce hypervirulent states [135]. Damage to the health infrastructure during these times results in inadequate sanitation and hygiene and limited access to healthcare services [136]. In the first-described disaster-associated outbreak, eight cases of *R. arrhizus* infection were reported following a volcanic eruption near Armero, Colombia, in 1985 [137]. These mostly involved severe necrotising fasciitis complicating trauma and carried a mortality of 75%. Notably, *R. arrhizus* was also isolated from the environmental culture of the disaster mud flow. Three cases of cutaneous mucormycosis from severe trauma (one due to *Mucor* spp. and two to *Apophysomyces elegans*) were also reported following the Indian Ocean Tsunami in 2004 [138,139,140].

A cluster of 13 cases of cutaneous mucormycosis in immunocompetent hosts was identified following an EF-5 tornado disaster in Joplin, Missouri, USA, in 2011 [141]. *Apophysomyces trapeziformis* was isolated from the wounds and other body sites of the patients, five of whom died (38.5%). It has been postulated that the high relative incidence of mucormycosis may be related to induction of hypervirulent phenotypes by the tornadic force [142]. Wurster et al. demonstrated that mechanical stress induced by magnetic stirring induced a hypervirulent phenotype for Mucorales within a *Drosophila* infection model [142].

Four ROCM infections due to *R. arrhizus* were reported after widespread flooding in Colorado, USA, in 2013 [143]. Each patient had risk factors for mucormycosis, including haematological malignancy and poorly controlled diabetes. Two patients died despite appropriate surgical and medical care. In another case series of invasive mould infections following floodwater damage from Hurricane Harvey in Houston, Texas, USA, in 2017, 5/20 cases were due to Mucorales infection [144]; however, there was not a selective predominance of causative genera, with the incidence of Mucorales, *Aspergillus*, and *Fusarium* invasive infections all increasing. Overall, while data are limited to case series, collectively, there is a plausible link between natural disasters and mucormycosis outbreaks, likely due to environmental disturbance, traumatic injury, suboptimal hygiene, and health infrastructure disruption. 

## 4. Diagnostics

The diagnosis of mucormycosis is ever challenging due to the variety of disease manifestations that often mimic other fungal infections, most notably invasive aspergillosis (IA). Diagnostic approaches must incorporate a thorough assessment, with a consistent clinical syndrome, alongside integration of radiological, histopathological, and microbiological findings (Figure 1).

### 4.1. Radiology

Radiographic findings in mucormycosis vary depending on the site of disease, and imaging often serves to establish clinical suspicion and to guide biopsy for definitive diagnosis [145]. For instance, a reverse halo sign or multiple nodules on chest computed tomographic (CT) imaging are characteristic signs of pulmonary mucormycosis [145]. Cranial CT or magnetic resonance imaging (MRI) findings in ROCM can be non-specific, as the most common finding of sinusitis can also represent disease caused by bacteria or other fungi, and follow-up endoscopic biopsy is strongly recommended [3]. CT or MRI angiography can assist in the assessment of angioinvasion and diagnosis of mycotic aneurysms [146,147,148]. There is also an emerging role of 18-Fluorodeoxyglucose Positron Emission Tomography (FDG PET) in diagnosis, staging of dissemination, and follow-up of mucormycosis [149,150,151]. 

### 4.2. Microscopy and Histology

Direct microscopic examination of clinical specimens, using a fluorescing dye such as Calcofluor white, can provide a rapid presumptive diagnosis of mucormycosis [3,152]. Suggestive findings of direct microscopy, even in the absence of a positive culture, should be considered significant [152]. The visualisation of characteristic mucormycete hyphae in tissue biopsy is essential to a definitive diagnosis [8]. Such features include findings of aseptate or pauciseptate ribbon-like hyphae (width 6–25 μm) with irregular branching, visualised by haematoxylin and eosin (H&E), periodic acid-Schiff (PAS), or Grocott–Gomori methenamine silver (GMS) staining. Importantly, histopathology alone cannot always distinguish between mucormycosis and other fungal infections [153]. While Mucorales are historically described as having right-angle branching as opposed to the acute-angle branching angles of septate moulds such as *Aspergillus*, this morphological feature can be masked by pressure effects on the fungus and the distorting effects induced by tissue processing [3].

Immunohistochemical (IHC) targets, such as α-1,6-mannan, have been investigated with limited in vitro success, as cross-reactivity with *Aspergillus*, *Fusarium* and *Candida* spp. limits their specificity for mucormycosis [154]. Monoclonal antibodies against specific Mucorales demonstrate high sensitivity and specificity, making them useful to differentiate mucormycosis against other invasive fungal infections, although they are not widely available [155,156,157]. 

### 4.3. Culture-Based Diagnostics

The culture of appropriate specimens from patients with suspected mucormycosis is essential to yield an isolate for accurate speciation and antimicrobial susceptibility testing [3]. However, sensitivity of culture in microscopically positive samples is only up to 50% [158]. It is critical that tissue samples are handled without homogenisation or grinding, which disrupts the hyphae and culture viability [159]. Morphological identification of Mucorales is detailed elsewhere [152,160]. Whilst morphological examination is key, other identification methods, such as proteomics or sequencing, are increasingly used. 

The case-series data on matrix-assisted laser desorption/ionization–time of flight (MALDI-TOF) mass spectrometry for Mucorales identification are promising [161,162], although molecular approaches remain the definitive method for species identification. The internal transcribed spacer regions (ITS1 and ITS2) of rDNA or the 18*S* or 28*S* rRNA genes themselves are the most frequently used targets, with the ITS region being the preferred DNA barcoding marker for fungal speciation [163,164]. To determine species identification, sequences are typically compared against the Westerdijk Fungal Biodiversity Institute (http://www.wi.knaw.nl/, accessed on 1 May 2023) and the International Society for Human and Animal Mycology (ISHAM) (http://its.mycologylab.org/, accessed on 1 May 2023) databases. However, as for many other fungal pathogens, a single target has not been identified that performs consistently well for speciation of all Mucorales [159].

### 4.4. Molecular Diagnostics

Molecular-based assays are pivotal in mucormycosis diagnosis, allowing for expedited results from clinical specimens and/or detection of Mucorales DNA in culture-negative specimens, as well as playing an emerging role in molecular biomarker monitoring. Polymerase chain reaction (PCR) diagnostics from tissue and bronchoalveolar lavage (BAL) fluid are well established, and fresh tissues in which fungal elements have been visualised carry higher diagnostic utility [165,166].

The spore-coating protein homolog encoding CotH genes has emerged as a promising PCR target, as the genes are uniquely and universally present among Mucorales [167,168]. Recent studies on a single-target CotH PCR assay demonstrated a sensitivity of 90% and a specificity of 100% in a mouse model of mucormycosis, with no CotH amplification seen in BAL fluid, plasma, and urine samples from uninfected and *Aspergillus*-infected mice [169]. Interestingly, CotH positivity was observed in urine, plasma, and BAL fluid from 24 h after infection, suggesting that this target may be useful for early diagnosis [169].

The diagnostic yield of PCR on serum or whole blood is limited; hence, the reported efficacy of Mucorales-specific PCR assays on these specimens varies widely. Assays incorporating multiple targets to encompass *Mucor*, *Rhizopus*, *Rhizomucor*, and *Lichtheimia* species have been used to diagnose early infection and to prognosticate based on treatment response [170]. In one study including 232 patients with suspected invasive mould disease, the PCR on whole blood was helpful in detecting mucormycosis a median of four days prior to culture positivity, and PCR negativity after amphotericin initiation was associated with an 85% reduction in 30-day mortality (adjusted hazard ratio, 0.14; 95% CI, 0.03–0.72; *p* = 0.02) [171]. An interlaboratory evaluation of this assay demonstrated high reproducibility and performance [172].

The commercial assay, MucorGenius^®^ (PathoNostics, Maastricht, The Netherlands), a semi-quantitative PCR assay targeting the 28*S* rRNA target, has shown modest sensitivity (75%) based on retrospective testing of 106 stored residual blood samples from 16 patients with culture-confirmed mucormycosis [173]. However, a positive MucorGenius^®^ PCR result preceded culture positivity by a median of eight days, again underlining the potential for the use of this assay in enabling early diagnosis [173]. A retrospective French study investigating pulmonary mucormycosis also demonstrated high sensitivity and specificity for both the MucorGenius^®^ and an in-house PCR [174]. By contrast, a Mucorales-specific semi-nested PCR on serum was not effective in the diagnosis of confirmed Mucorales-associated sinusitis in immunocompromised patients where none of the five patients was identified [175].

Isothermal amplification methods are not in routine use for the diagnosis of mucormycosis. That being said, one report using 42 reference strains of Mucorales suggested the utility of this approach as a sensitive and specific diagnostic tool for rapid diagnosis of *R. microsporus*, *R. arrhizus* var. *arrhizus*, *R. arrhizus* var. *delemar*, *M. irregularis*, *M. circinelloides*, *L. ramosa,* and *L. corymbifera* infections [176]. A high-resolution melting analysis (HRMA) of PCR products from BAL fluid also showed promise in differentiating some Mucorales species with a sensitivity of 100% and specificity of 93% [177].

### 4.5. Serological Diagnosis

Antigen-based diagnostic assays in mainstream use for invasive fungal infections—namely the galactomannan and beta-d-glucan assays—are not useful in the diagnosis of mucormycosis due to the absence of these antigens in the cell walls of Mucorales [178]. The possibility of dual infections must be considered, particularly in immunocompromised patients, so a positive galactomannan or beta-d-glucan result does not necessarily exclude concurrent mucormycosis. Early studies of novel antigen targets have shown promise, such as the use of the *Rhizopus*-specific antigen for diagnosis of *R. arrhizus* infection in murine models [179].

Several serological assays have been evaluated for the diagnosis of mucormycosis, using ELISA-based techniques, Western immunoblots, and immunodiffusion assays. ELISA-based assays have displayed sensitivities of 81% and specificities of 97% but demonstrated non-specific cross-reactivity with *Candida* and *Aspergillus* spp., using serum samples [180]. None of these approaches has accumulated sufficient clinical data to support clinical implementation [155,179,181]. Lateral flow immunoassays (LFIAs), unlike for IA [182], are not yet of utility in the diagnosis of mucormycosis; however, initial reports of an LFIA targeting fucomannan, a Mucorales cell-wall carbohydrate, have shown promising results on serum, urine, and BAL fluid in a murine model [183].

One novel approach under investigation for the diagnosis of mucormycosis is an enzyme-linked immunospot (ELISpot) or immunocytofluorimetric assay, which detects Mucorales-specific T-cell activity via quantitation of interferon-gamma release from activated T cells [184,185,186]. The preliminary observational data are promising, but clinical data are limited to very small case series.

### 4.6. Emerging Diagnostic Approaches

Due to the clinical severity of invasive mucormycosis and association of delayed diagnosis with compromised clinical outcomes [187], emerging diagnostic techniques are constantly under evaluation to improve the timeliness and accuracy of diagnosis. Research is ongoing into the utility of metabolomic signatures such as breath tests for volatile metabolites [188], biosensor-based approaches in clinical specimens [189], fluorescence in situ hybridisation techniques for diagnosis from formalin-fixed paraffin-embedded tissues [190], and mass spectrometry of serum disaccharides [191]. To date, these methodologies remain under active exploration within the research sphere only.

### 4.7. Susceptibility Testing

The European Committee on Antimicrobial Susceptibility Testing (EUCAST) and the Clinical and Laboratory Standards Institute (CLSI) have developed standardised methodologies for antifungal susceptibility testing of Mucorales [192,193]. While the methods differ, there is good agreement, although EUCAST minimum inhibitory concentrations (MICs) tend to be 1- to 3-fold dilutions higher than those of CLSI [194,195]. Spectrophotometric readings show reasonable agreement with visual inspection of microdilution plates [196,197,198]. Epidemiological cutoff values (ECVs or ECOFFs) have been determined for some species, but clinical breakpoints (CBPs) have not been defined. Due to species-specific variations in antifungal susceptibility, in vitro antifungal susceptibility testing is recommended to establish epidemiologic knowledge to aid in the establishment of ECVs/ECOFFs and CBPs (Table 3), as well as to ultimately optimise treatment [199].

In vitro MICs show that amphotericin B is the most active antifungal against most Mucorales [201,202,203,204,205]. In one small series of 10 mucormycosis cases, there was a significantly better 6-week response when the pathogen MIC was ≤0.5 mg/L versus >0.5 mg/L for amphotericin B (83% versus 0%; *p* = 0.05) [206]. The ≥95% ECV for amphotericin B against *L. corymbifera*, *M. circinelloides*, *R. arrhizus*, and *R. microsporus* was calculated to be 1, 1, 2, and 2 mg/L, respectively (Table 3) [200]. Mucorales, which exhibit higher MICs for amphotericin B, include *Apophysomyces* spp. and *Cunninghamella bertholletiae* [200,202,203,204,207].

Posaconazole and isavuconazole have good in vitro activity against many Mucorales. The 95% ECV of posaconazole for *L. corymbifera*, *R. arrhizus*, and *R. microsporus* is 1 mg/L, while the ECV for *M. circinelloides* is 4 mg/L (Table 3) [200]. While posaconazole has overall lower MICs than isavuconazole, its blood concentrations are lower, and the clinical significance of these differences are unclear [195,204,208]. *Rhizopus* spp. tend to have higher posaconazole MICs, while *M. circinelloides* tends to have higher isavuconazole MICs [195,203,205,209]. Itraconazole activity is variable and more species specific (Table 3). Other triazoles, including voriconazole, and echinocandins display high MICs against Mucorales, and are considered intrinsically resistant [202,203,210,211]. While terbinafine is active in vitro against some Mucorales (geometric mean MIC < 1 mg/L for *L. corymbifera*, *Cunninghamella* spp., *R. microsporus*, and *R. pusillus*), its efficacy is poor in animal models [199,203].

There have been a small number of studies looking at the synergistic activity of antifungal combinations using chequerboard microdilution methods [212]. Certain drug combinations have synergistic activity against some strains and species, but this is not readily predictable [213,214,215,216]. Dannaoui et al. studied 35 Mucorales isolates and found combination amphotericin B/rifampicin to be synergistic or additive, combination amphotericin B/terbinafine to be synergistic for 20% of isolates, and combination terbinafine/voriconazole to be synergistic for 44% of isolates [213]. On the other hand, there was neither synergism nor antagonism with combination amphotericin B/posaconazole amongst 11 *R. arrhizus* isolates [215]. Schwarz et al. reviewed combination antifungal therapy for mucormycosis, outlining existing studies for combination susceptibility testing for both antifungal and non-antifungal drugs and found that while antagonism is rare, synergy rates were highly variable [212].

A limited number of studies have compared the reference broth microdilution method against alternative methods such as disc diffusion or gradient strip testing. Disc diffusion appears feasible, with a low rate of major and very major errors demonstrated for posaconazole and amphotericin B when using cutoffs of 1 and 4 mg/L to pragmatically define sensitivity and resistance, respectively [207]. There have been conflicting studies with gradient strip testing, with some studies showing reasonable agreement of MICs and others demonstrating conflicting results, especially for amphotericin B and posaconazole [197,217,218,219].

Molecular determinants of antifungal resistance are not well studied or described. Research into *R. arrhizus* has demonstrated that CYP51A is also responsible for its intrinsic voriconazole and fluconazole resistance [220,221]. Modelling studies suggest that the differential activity of the azoles against *Rhizopus* and *Mucor* spp. may be explained by a key F129 residue of the CYP51 F5 gene in Mucorales spp. which confers decreased binding of short-side-chain (fluconazole, voriconazole) but not long-side-chain azoles (posaconazole) [222].

### 4.8. Whole-Genome Sequencing

Earlier investigations of mucormycosis case clusters have detailed epidemiological linkages without data on the genetic relatedness between strains, partly as there are no established genotyping makers for Mucorales [105,132]. The advent of WGS approaches for phylogenetic analyses has yielded new information, as well as challenges in delineating genetic diversity within different species [128,129,130,131,141,223]. Firstly, a WGS analysis of Mucorales is problematic due to the large size of their genomes (45.3 Mbp for *R. arrhizus*), as well as the presence of whole-genome duplication events [221]. Most assembled genomes of *R. microsporus* var. *rhizopodiformis* strains were ≅25 Mbp, but some isolates had larger genomes (43–51 Mbp), which are indicative of genome expansion [130]. The genomes of *R. pusillis* (≅25 Mbp), *Apophysomyces* (≅32 Mbp), and *Lichtheimia* spp. (≅58 Mbp) are larger still [128,224]. Genome size influences pre-analytic and analytic factors such as capacity, throughput, selection of sequencing coverage, and read depths.

Secondly, reference genomes for Mucorales are less rigorously standardized in comparison with reference bacterial genomes. Nonetheless, there are publicly available reference genomes that have been assembled by research groups, such as *M. circinelloides* 1006 PhL [114], *M. indicus* B7402 [225,226], *R. microsporus* (GenBank: GCA_006680115.1) [130], and *R. pusillus* (GenBank: GCA_900175165.2) [128]. Variability of comparative sequence results may be evident with the use of different reference genomes. Thirdly, deriving the genetic threshold or pairwise distance cutoff to determine if two or more strains within a species are clonal can be problematic. This threshold will vary with species, recombination events, and the bioinformatics pipeline used. In addition, the genome variability of Mucorales within the hospital environment and in the community is uncertain in comparison to baseline genomic epidemiology, which is fundamental to contextualising the relatedness of strains [125].

Garcia-Hermoso et al. were amongst the first to use WGS analysis to investigate a possible nosocomial cluster of six patients with *Mucor circinelloides* sensu stricto wound infection [129]. Twenty-one isolates (fourteen outbreak and seven unrelated isolates) were studied against the reference genome *M. circinelloides* 1006PhL. This study set the premise that the genome of *M. circinelloides* was stable, established parameters of the sequencing process, and assigned a genetic threshold (by estimating evolutionary distance and number of single nucleotide polymorphisms (SNPs) between pairs of isolates) to determine the genetic proximity between genomes from identical or clonal strains. Unexpectedly, approximately 5296 SNPs were identified as a cutoff below which two isolates could be designated as being of the same strain. Four clades were identified, where each clade was separated by >290,000 SNPs, while isolates within each clade varied by ≅20,000 SNPs [223,227]. Contrary to the initial notion that single-strain clonal transmission caused the outbreak, the cluster was due to multiple unrelated strains present in the environment. These conclusions are similar to those of the Joplin outbreak investigation [141,224] where there was no linkage between genetic groups of *A. trapeziformis* and exposure [224].

In a WGS-directed investigation of three cases of mucormycosis in heart and lung transplant patients over 6 months, genome assembly of two *R. pusillus* strains yielded > 5900 core SNPs between them; the third isolate was *Lichtheimia ramosa* [128]. This report reiterated the need to interpret core SNPs between isolates with caution in the absence of defined cutoffs for clonal vs. non-clonal strains. For *R. microsporus* var. *rhizopodiformis*, however, 21 of 24 isolates associated with a cluster of surgical infections were of a single clade, with 17 of the 21 forming an inner clade consisting of only 1235 SNPs (range 60–912, mean 430). Although this is indicative of a likely common geographic origin, the genomic diversity was reported to be inconsistent with a point source outbreak [130].

Similarly, Nguyen et al. investigated a cluster of four transplant-associated cases of mucormycosis (three *Rhizopus* spp. and one *Lichtheimia* sp.) over a 10-month period and performed WGS on 72 clinical and environmental Mucorales including the outbreak isolates [131]. Both core and pan-genome analyses were performed. Notably, the core genome analysis had insufficient resolution to identify unique isolates and falsely clustered them. The pangenome analysis indicated extensive genetic diversity among clinical and environmental isolates.

Thus, while WGS approaches can resolve genetic diversity between strains, there remain challenges in demonstrating definitive associations with clinical and environmental strains, or to pinpoint a source of infection. Defining thresholds of SNP variation between isolates to determine relatedness remains challenging.

## 5. Management and New Antifungal Agents

**Figure 2:**
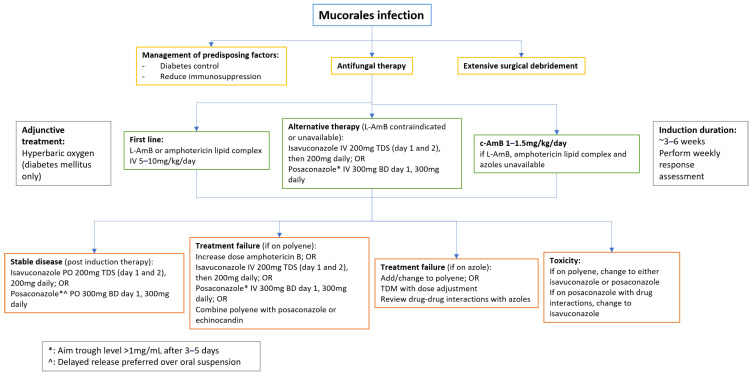
Algorithm for Management of Mucormycosis (adapted from [3,153,228,229]). L-AmB, Liposomal Amphotericin B; c-AmB, Amphotericin B deoxycholate.

Optimal management of Mucorales infection remains challenging, as there is a scarcity of randomised clinical trials to direct best practice treatment, and management guidelines are based on open-label studies, small clinical case series, and expert opinion [3]. The European Confederation for Medical Mycology (ECMM), in conjunction with the American Society for Microbiology (ASM) and International Society for Human and Animal Mycology (ISHAM), recommends a multimodal approach including (i) aggressive surgical debridement or resection of the affected region(s), (ii) antifungal therapy, and (iii) strict management of predisposing factors such as uncontrolled diabetes mellitus and neutropenia [3]. More recently, analogous recommendations have been made for the treatment of CAM [153,228]. The current treatment recommendations are summarised in Figure 2.

The preferred agent for empiric antifungal therapy, as well as targeted treatment in both adult and paediatric patients, is high-dose amphotericin B, with improved treatment success achieved with lipid amphotericin B formulations (either liposomal or lipid complex), while the deoxycholate formulation is recommended only in cases where other endorsed amphotericin B formulations are unavailable. Isavuconazole and posaconazole (delayed release tablets and intravenous formulations only) are both reasonable first-line alternatives when amphotericin is contraindicated, or they can alternatively be used as salvage therapy. Both are preferred over posaconazole oral suspension, as the latter is associated with higher rates of treatment failure [3,229]. When posaconazole is used, routine therapeutic drug monitoring (TDM) is strongly recommended, aiming for a trough level of >1 mg/L, while there is no current evidence to suggest that TDM is required for isavuconazole [3].

Combination antifungal therapy has been studied. For treatment refractory cases, the evidence suggests improved outcomes with combined amphotericin and echinocandin, or amphotericin and posaconazole; however, other retrospective evaluations report treatment equivalence, particularly in first line therapy [212]. Thus, this strategy is usually reserved for salvage therapy and in severely immunocompromised individuals [230]. Hyperbaric oxygen exposure in individuals with diabetes has been recommended as adjunctive therapy, with success described in several case reports and series in this population [230].

The treatment arsenal for Mucorales infection remains limited, and, even with current best-practice therapy, mortality rates over 60% are frequently described. Furthermore, current antifungal agents have limitations in terms of substantive toxicity and/or variable in vitro activity [231]. Thus, alternative therapeutic options are needed, and they are the subject of investigation. New antifungal agents, some with novel modes of action, are under varying stages of development and clinical evaluation for treatment of Mucorales infection. Those that have gained more recent attention include oteseconazole, manogepix (and its prodrug fosmanogepix), and jawsamycin.

Oteseconazole (VT-1161; Viamet Pharmaceuticals Inc., Durham, NC, UK), a long side-chain azole, is a novel metalloenzyme inhibitor which prevents ergosterol synthesis through selective inhibition of fungal CYP51 (lanosterol 14α-demethylase) [232]. Early studies noted potency in vitro against *R. arrhizus* var. *arrhizus*; however, the potency against *R. arrhizus* var. *delemar* was poor. In vivo *R. arrhizus* var. *arrhizus* infection studies in immunocompromised mice demonstrated a ≅1 log decrease in lung and brain fungal burdens in treated mice compared to placebo controls [233,234]. Whilst clinical trials of oteseconazole are in progress for other fungal infections, such as vulvovaginal candidiasis, further data on its efficacy in humans for either prophylaxis or treatment of Mucorales infections are needed.

Fosmanogepix (prodrug of manogepix) prevents the maturation of glycerolphosphatidyl inositol (GPI)-anchored proteins by inhibiting the enzymatic action of inositol acyltransferases (Gwt1), compromising fungal growth. In a murine model, combined fosmanogepix with liposomal amphotericin B was superior to amphotericin B alone in the treatment of invasive mucormycosis [235]. Potent in vitro profiles were exhibited in both *R. arrhzius* var *arrhizus* and *R. arrhizus* var. *delemar* isolates, and efficacy has been demonstrated when used as a single agent to treat *R. arrhizus* pulmonary and disseminated infection in immunosuppressed mice [236]. Clinical-outcome data in humans are currently absent; a phase II clinical trial (NTC04240886) was terminated prematurely due to enrolment issues. A phase III study has been planned.

Jawsamycin (FR-900848) inhibits the Spt14/Gpi3-mediated biosynthesis of glycosylphosphatidylinositol, which is essential to maintain the integrity of the fungal cell wall. Low MICs were recorded when ATCC isolates of six common species of Mucorales were exposed to jawsamycin in vitro, including *R. arrhizus*, *L. corymbifera*, and *M. circinelloides.* Additionally, enhanced survival and a decreased fungal burden were demonstrated in murine models with pulmonary infection due to *R. arrzhius* var. *delemar* [237].

Calcineurin and mTOR inhibitors are more novel treatment options which have both demonstrated synergistic in vitro activity against Mucorales isolates when combined with posaconazole, isavuconazole, or amphotericin B [238,239,240]. In vivo efficacy has been shown with rapamycin alone (in an insect model) and tacrolimus in combination with posaconazole (in insect and murine models) [241,242].

There are several other novel antifungal agents in early stages of evaluation against Mucorales, with some promising in vitro and in vivo activity, particularly for *Rhizopus* spp. These agents are detailed in Table 4. At the time of writing, there are no proposed clinical trials or clinical evaluations in humans.

## 6. Conclusions

In this paper, we described significant advances in diagnostic methods and promising signals for new antifungal therapy options, but significant challenges still exist in regard to controlling the burden of disease caused by this WHO high-priority pathogen group. First, an accurate estimate of the burden is required, along with a better understanding of the epidemiology of disease—especially as at-risk populations continue to emerge and expand. This requires systematic surveillance across countries, using common data points and ensuring that laboratory data are married to clinical meta-data.

Research and development efforts must focus on developing accurate, cheap, rapid diagnostic tests—ideally available in a point-of-care format. Advanced molecular epidemiology is complicated and challenging but holds significant promise; however, robust pathology stewardship will be needed.

Research must continue to focus on the development of new antifungal agents and adjunctive therapies. Mortality is unacceptably high with the current armamentarium, and newer agents, while promising, still require further investigation before being introduced to routine clinical care. Finally, there must be equitable access to available treatments and diagnostics. Improved supply chains, fair pricing, and capacity building will contribute to this goal.

## Figures and Tables

**Table 1:** Clinically relevant species of Mucorales with their modern assignments in the context of their family structure (as published by Hoffman et al. [16] and adapted from Walther et al. [15]).

Family	Genus	Clinically Relevant Species
Mucoraceae	*Actinomucor*	*A. elegans*
Saksenaeaceae	*Apophysomyces*	*A. mexicanus* *A. ossiformis* *A. trapeziformis* *A. variabilis*
Mucoraceae	*Cokeromyces*	*C. recurvatus*
Cunninghamellaceae	*Cunninghamella*	*C. arunalokei* [20] *C. bertholletiae* *C. blakesleeana* *C. echinulata* *C. elegans*
Lichtheimiaceae	*Lichtheimia*	*L. corymbifera* *L. ornata* *L. ramosa*
Mucoraceae	*Mucor*	*M. amphibiorum* *M. circinelloides* * M. griseocyanus * M. indicus *M. irregularis* *M. janssenii* * M. lusitanicus * M. plumbeus *M. racemosus* *M. ramosissimus* * M. variicolumellatus * M. velutinosus *
Lichtheimiaceae	*Rhizomucor*	*R. miehei* *R. pusillus*
Rhizopodaceae	*Rhizopus*	*R. arrhizus* (including var. *arrhizus* and var. *delemar*) *R. homothallicus* *R. microsporus* *R. schipperae*
Saksenaeaceae	*Saksenaea*	*S. erythrospora* *S. loutrophoriformis* *S. trapezispora* *S. vasiformis*
Syncephalastraceae	*Syncephalastrum*	*S. racemosum*
Lichtheimiaceae	*Thamnostylum*	*T. lucknowense*

* Member of the *Mucor circinelloides* complex [18].

**Table 2:** Classical host factors with associated clinical syndromes of mucormycosis.

Host Factor	Associated Clinical Syndrome	References
Diabetes mellitus, particularly with ketoacidosis	ROCM	[67]
Corticosteroid use	ROCM	[68]
Haematologic malignancies	Pulmonary or disseminated infection	[68]
COVID-19	ROCM	[6]
Haematopoietic cell transplantation	Pulmonary	[69]
Solid organ transplantation	Disseminated infection	[70]
HIV/AIDS	Disseminated infection	[71]
Treatment with deferoxamine	ROCM	[72]
Iron overload	ROCM	[73]
Injection drug use	Isolated cerebral	[74]
Major trauma	Cutaneous	[75]
Burns	Cutaneous	[34]

**Table 3:** Epidemiologic cutoff values (ECVs) of minimum inhibitory concentrations (MICs) of amphotericin B (AMB), posaconazole (POS), and itraconazole (ITR) and for four Mucorales species (adapted from [200]).

Species	Antifungal	MIC (mg/L)		Calculated ECV	
		Range	Mode	≥95%	≥97.5%
*L. corymbifera*	AMB	0.06–16	0.5	1	2
	POS	0.06–4	0.5	1	2
	ITR	0.06–8	0.25	Not determined	
*M. circinelloides*	AMB	0.03–4	0.25	1	2
	POS	0.06–16	1	4	4
	ITR	0.25–16	4	Not determined	
*R. arrhizus*	AMB	0.03–4	1	2	4
	POS	0.03–32	0.5	1	2
	ITR	0.06–16	0.5	2	2
*R. microsporus*	AMB	0.06–4	0.5	2	2
	POS	0.06–16	0.5	1	2
	ITR	0.25–32	1	Not determined	

**Table 4:** New and novel antifungal agents with in vitro and/or in vivo activity against Mucorales.

Agent	Mechanism of Action	Efficacy	References
Opelconazole (inhaled)	Inhibition of formation of ergosterol in fungal cell membrane	In vitro activity against *R. arrhizus* (syn. *oryzae)* (ATCC 11145) (MIC 2 mg/L); poor activity against *R. pusillus, M. circinelloides,* and *L. corymbifera*	[243]
AR-12	Celecoxib derivative which inhibits fungal acetyl coenzyme A (acetyl-CoA) synthetase	In vitro activity against *R. arrhizus* (MIC 4 mg/L)	[244]
MGCD290	Inhibits fungal histone deacetylase 2	In vitro synergy with triazoles	[245]
Statins (fluvastatin, rosuvastatin or atorvastatin)	Affect the synthesis of ergosterol by inhibiting 3-hydroxy-3-methylglutaryl-CoA (HMGCoA) reductase	In vitro synergy with either amphotericin B or various azoles in combination	[246,247]
Anti-CotH3 antibodies	Monoclonal or polyclonal antibodies that inhibit CotH3, a fungal cell protein which binds to glucose regulated protein 78 on endothelial cells	In vivo activity in murine models with improved outcomes.	[248]
Colistin	Polymyxin antibiotic	In vitro and in vivo activity in murine models Single case report of GI mucormycosis demonstrated treatment success when used in combination with standard of care	[249,250]
Silver nanoparticles	Small particles with known antimicrobial activity	Potent in vitro activity against some *R. arrhizus* isolates (MIC < 8–64 mg/L)	[251]
Sofosbuvir	Antiviral targeting RNA-dependent RNA polymerase	In silico experiments demonstrating inhibition of *R. arrhizus*	[252]

## Data Availability

Not applicable.

## Abbreviations

AMB:	Amphotericin B
ATCC:	American Type Culture Collection
BAL:	Bronchoalveolar lavage
c-AmB:	Amphotericin B deoxycholate
CAM:	COVID-19-associated mucormycosis
CBP:	Clinical breakpoint
CI:	Confidence interval
CLSI:	Clinical and Laboratory Standards Institute
CT:	Computed tomography
COVID-19:	Coronavirus disease 2019
DNA:	Deoxyribonucleic acid
ECV/ECOFF:	Epidemiological cutoff value
EUCAST:	European Committee on Antimicrobial Susceptibility Testing
FDG-PET:	Fluorodeoxyglucose positron emission tomography
FFPE:	Formalin fixed paraffin-embedded
GI:	Gastrointestinal
GMS:	Grocott–Gomori methenamine silver
H&E:	Haematoxylin and eosin
HRMA:	High-resolution melting analysis
IA:	Invasive aspergillosis
ICU:	Intensive care unit
IHC:	Immunohistochemistry
ITS:	Internal transcribed spacer
L-AmB:	Liposomal Amphotericin B
LFIA:	Lateral flow immunoassay
MALDI TOF	Matrix assisted laser desorption ionisation-time of flight
MIC:	Minimum inhibitory concentration
MRI:	Magnetic Resonance Imaging
PAS:	Periodic acid-Schiff
PCR:	Polymerase chain reaction
POS:	Posaconazole
RNA:	Ribonucleic acid
ROCM:	Rhino-orbital-cerebral mucormycosis
SNP:	Single nucleotide polymorphism
TDM:	Therapeutic drug monitoring
ITR:	Itraconazole
WGS:	Whole genome sequencing
